# Flying south: Foraging locations of the Hutton's shearwater (*Puffinus huttoni*) revealed by Time‐Depth Recorders and GPS tracking

**DOI:** 10.1002/ece3.5171

**Published:** 2019-06-28

**Authors:** Della G. Bennet, Travis W. Horton, Sharyn J. Goldstien, Lindsay Rowe, James V. Briskie

**Affiliations:** ^1^ School of Biological Sciences University of Canterbury Christchurch New Zealand; ^2^ Department of Geological Sciences University of Canterbury Christchurch New Zealand; ^3^ Hutton's Shearwater Charitable Trust Kaikōura New Zealand

**Keywords:** Global Positioning Systems, Hutton's Shearwater, New Zealand, *Puffinus huttoni*, Time‐Depth Recorders

## Abstract

1. The Hutton's shearwater *Puffinus huttoni* is an endangered seabird endemic to Kaikōura, New Zealand, but the spatial and temporal aspects of its at‐sea foraging behavior are not well known.
2. To identify foraging areas and estimate trip durations, we deployed Global Positioning Systems (GPS) devices and Time‐Depth Recorders (TDR) on 26 adult Hutton's shearwaters during the chick‐rearing period in 2017 and 2018.
3. We found Hutton's shearwaters traveled much further from their breeding grounds at Kaikōura than previously considered, with most individuals foraging in coastal and oceanic areas 125–365 km south and near Banks Peninsula. Trip durations varied from 1 to 15 days (mean = 5 days), and total track lengths varied from 264 to 2,157 km (mean = 1092.9 km).
4. Although some diving occurred in near‐shore waters near the breeding colony, most foraging was concentrated in four regions south of Kaikōura. Dive durations averaged 23.2 s (range 8.1 to 71.3 s) and dive depths averaged 7.1 m (range 1.5 to 30 m). Foraging locations had higher chlorophyll *a* levels and shallower water depths than nonforaging locations. Birds did not feed at night, but tended to raft in areas with deeper water than foraging locations.
5. Mapping the spatial and temporal distribution of Hutton's shearwaters at sea will be fundamental to their conservation, as it can reveal potential areas of overlap with fisheries and other industrial users of the marine environment.

## 1 INTRODUCTION

Seabirds are one of the most threatened groups of marine species (Croxall et al., 2012). As anthropogenic activities increase, conflicts between seabirds and tourism, fisheries, and oil exploration are increasing (Uruski, 2010; Markowitz, Richter, & Gordon, 2011; Richard, Abraham, & Filippi, 2015). Until recently, the difficulty of studying the at‐sea behavior of seabirds limited our ability to manage these conflicts. With recent improvements in accuracy and reduction in size of Global Positioning Systems (GPS), and Time‐Depth Recorders (TDR), it is now possible to track even some of the smallest species of seabirds, making the study of their behaviors more practical (Freeman et al., 2010; Navarro et al., 2013). Especially for threatened species, mapping the movements of birds at sea is a key first step in understanding the impacts of human activity, aiding in the establishment of marine protection areas and minimizing seabird–fisheries interactions (Croxall et al., 2012).

While at sea, some seabirds are known to travel large distances to reach profitable foraging sites (Jodice & Suryan, 2010), but during the breeding season, most species can be classified as central place foragers, returning regularly to breeding colonies to incubate or feed their chick. However, variation in productivity and foraging conditions can affect the foraging behavior of a species, whereby some individuals either forage at great distances from the colony or remain close to the nesting site (Jaeger et al., 2014; Paiva, Pereira, Ceia, & Ramos, 2017). Such “bimodal” patterns of distribution at sea have been found in Cory's shearwater *Calonectris diomedea*, and sooty shearwater *Puffinus griseus* (Baduini & Hyrenbach, 2003; Paiva et al., 2017; Shaffer et al., 2009). Patterns of foraging can also change due to seasonal changes in environmental conditions (e.g., chlorophyll *a* levels), increased levels of competition, stochastic events, and greater fisheries pressures (Jodice & Suryan, 2010; Richard et al., 2015; Paiva et al., 2017).

**Figure 1:**
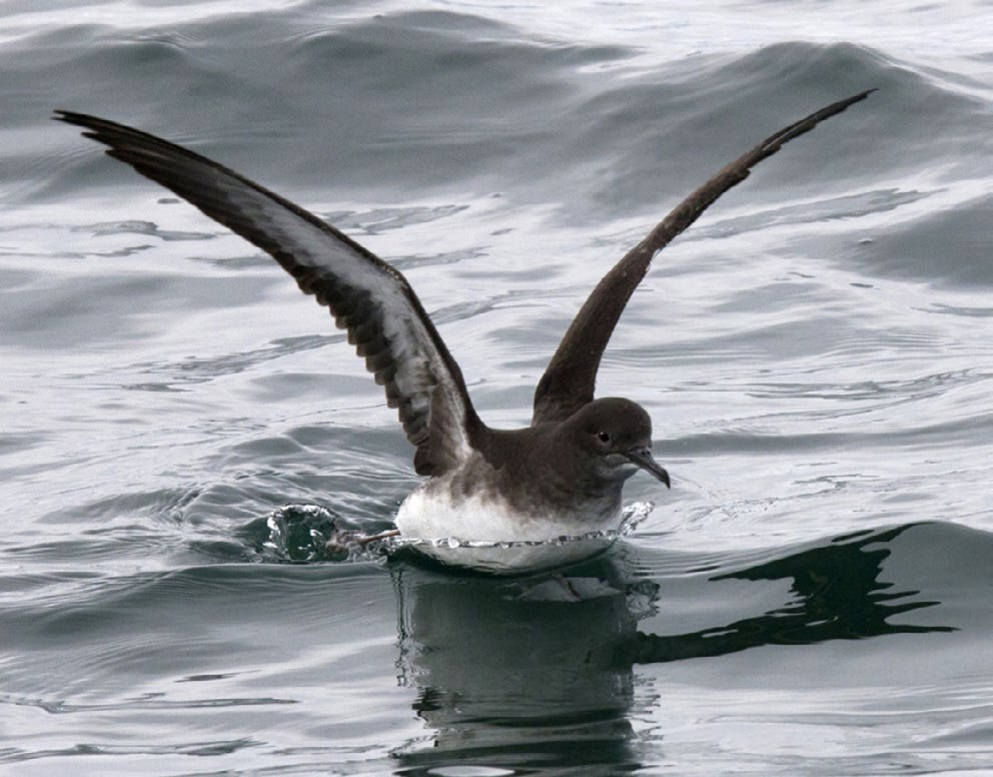
Hutton's shearwater about to take flight off the Kaikōura Peninsula, New Zealand, 20 September 2014 (photograph by Della Bennet)

The Hutton's shearwater *Puffinus huttoni* is a breeding seabird endemic to the Kaikōura region of New Zealand (Figure 1). Its breeding biology on land has been well studied (Cuthbert & Sommer, 2009; Sommer et al., 2009), but most observations of its at‐sea behavior are anecdotal (Taylor, 2000). For example, flocks of Hutton's shearwater have been reported along the Kaikōura and Canterbury coastline south to Banks Peninsula, out to the Chatham Rise in the east, and north to the Cook Strait (Harrow, 1976; Hawke, 1998; Pinkerton, 2011). These areas coincide with longline and trawl fisheries due to their high species richness (Francis, Hurst, McArdle, Bagley, & Anderson, 2002; Leathwick, Elith, Francis, Hastie, & Taylor, 2006; McClatchie et al., 1997; Richard et al., 2015). Although Hutton's shearwaters generally do not associate or follow boats (Marchant & Higgins, 1990; Wood, 1993), at least two by‐catch occurrences of Hutton's shearwaters have been reported in the region (Tarburton, 1981; West & Imber, 1985). Given the endangered status of Hutton's shearwater, a systematic assessment of its at‐sea activities is needed to understand more about their foraging ecology and distribution at sea, how susceptible they are to anthropogenic activity, and how they may be affected by shifts in ocean conditions (Cuthbert, 2001; Marchant & Higgins, 1990).

The objectives of this study were to: (a) describe the at‐sea movements of Hutton's shearwater through the deployment of GPS technology and (b) identify the foraging areas using TDRs. We then compared estimates of chlorophyll *a* and bathymetry at known dive locations with nonforaging areas to determine if the movements of Hutton's shearwaters correlated with local productivity. Our data provide the first systematic information on the foraging locations and at‐sea behavior of this endangered species, and a baseline for future mapping of the spatial and temporal distribution of Hutton's shearwaters at sea in response to changes in levels of human activity and oceanic conditions.

## 2 MATERIALS AND METHODS

### 2.1 GPS and TDR deployment

During January 2017 and January–February 2018, breeding Hutton's shearwater adults were captured from their nesting burrows within the recently established Kaikōura Peninsula colony (Te Rae o Atiu; −42.4286 S, 173.7029 E). For practical reasons, several different types of tracking devices were used (Table 1). Eight PinPoint50 Global Positioning System trackers (GPS; 22 × 13 × 9 mm, 2.2 g, Lotek Wireless), five Uria100 GPS trackers (35 × 16 × 11 mm, 8.5 g, Ecotone), and eight LAT1500 TDR (8 × 32 mm, 3.4 g, 512 kb memory, Lotek Wireless) were deployed on 23 birds during the 2017 chick‐rearing period. Both adults were fitted with trackers at 11 nests that contained a chick, but only one adult was tracked at a twelfth nest. During the 2018 chick‐rearing period, six PinPoint120 GPS standard trackers (39 × 13.1 × 11.7 mm, 5.0 g, Lotek Wireless), six PinPoint120 GPS Swift Fix trackers (24.6 × 16.4 × 15.5, 7.0 g, Lotek Wireless), and six LAT1500 TDRs were deployed on 16 birds. In 2018, adults from 14 nests were used for instrumentation, but both adults were tracked at only two nests. Only one adult from each pair was tracked at a time and redeployment on the nesting partner was not initiated until 1–2 days later. In both years, all birds had been banded and sexed previously, and individual identification was confirmed through the leg band number.

**Table 1:** Summary of deployments of GPS (2017 back and 2018 tail attachment), and Time‐Depth Recorders (TDR) data loggers attached to Hutton's shearwater adults in the Kaikōura Peninsula colony during the breeding seasons of 2017 and 2018

Year	Bird	Nest	Band	Sex	Body mass (g)	TDR	GPS		Date		Trip	
							Style	Fix rate (min)	Deployment	Recovery	Length (days)	Completion
2017	A	42	X16908	Female	315		U100	5	11‐Jan	20‐Jan	9	N (4 days 18 hr)
2017	B	77	X16926	Female	365		U100	5	11‐Jan	13‐Jan	2	Y
2017	C	45	X16963	Female	350	Y	PP50	10	11‐Jan	13‐Jan	2	N (6 hr)
2017	D	37	X19665	Male	340	Y	PP50	10	11‐Jan	16‐Jan	5	N (24 hr)
2017	E	40	X17261	Male	335		U100	5	12‐Jan	20‐Jan	8	N (3 days 22 hr)
2017	F	53	X17334	Male	345	Y	PP50	30	15‐Jan	24‐Jan	9	N (16 hr)
2017	G	90	X17105	Female	330		U100	5	16‐Jan	20‐Jan	4	N (3 days 10 hr)
2017	H	40	X17000	Female	330		U100	15	21‐Jan	25‐Jan	4	Y
2017	I	37	X16995	Female	320		U100	15	21‐Jan	25‐Jan	2	Y
2017	J	42	X16980	Male	320	Y	PP50	60	26‐Jan	28‐Jan	2	Y
2017	K	51	X17216	Male	435	Y	PP50	60	26‐Jan	10‐Feb	15	N (1 days 1 hr)
2018	A	N42	X16908	Female	327		PP120	60	22‐Jan	26‐Jan	4	N (3 days 20 hr)
2018	C	N45	X16963	Female	335		PP120	60	26‐Jan	30‐Jan	4	N (3 days 9 hr)
2018	D	N55	X19665	Male	327	Y^a^	Swift	15	24‐Jan	30‐Jan	6	Y
2018	E	N40	X17261	Male	335	Y	Swift	15	20‐Jan	26‐Jan	6	Y
2018	K	N51	X17216	Male	412		PP120	60	25‐Jan	29‐Jan	4	Y
2018	L	N46	X15997	Female	352		PP120	60	21‐Jan	23‐Jan	2	Y
2018	M	N45	X16917	Male	336	Y	Swift	15	21‐Jan	22‐Jan	1	Y
2018	N	N41	X16934	Male	338		PP120	60	24‐Jan	26‐Jan	2	N (1 day 15 hr)
2018	O	N24	X16961	Female	417		PP120	60	21‐Jan	24‐Jan	3	N (2 days 14 hr)
2018	P	N77	X17115	Male	390		PP120	60	23‐Jan	29‐Jan	6	N (5 days 21 hr)
2018	Q	N59	X17149	Female	338		PP120	60	24‐Jan	29‐Jan	5	N (1 day 15 hr)
2018	R	N72	X17154	Female	388	Y	Swift	15	20‐Jan	27‐Jan	7	Y
2018	S	N53	X19101	Female	348		Swift	15	20‐Jan	1‐Feb	11	Y
2018	T	N55	X19106	Female	344		PP120	60	21‐Jan	23‐Jan	2	N
2018	U	N38	X19706	Male	366	Y	Swift	15	21‐Jan	25‐Jan	4	Y

Note

Y, bird returned to the colony with a continuous GPS track and/or TDR unit deployed; N, GPS tracker battery depleted prior to returning to the colony; U100, Uria100; Ecotone GPS tracker; PP50 and PP120: PinPoint50 and PinPoint120, Lotek Wireless GPS Tracker; Swift: SwiftFix, BioTrack Wireless GPS tracker.

a TDR failed to record data.

To fit the tracking equipment, birds were caught by hand from within the artificial nesting boxes provided. The GPS tracker and TDR logger were prepared and software programs deployed before attachment. Birds were held within a black cotton bag to reduce stress and prevent biting. While bagged, adults were weighed prior to equipment attachment and after retrieval using a spring Pesola balance (±5 g). To avoid irritation from a chest harness, and to avoid restriction and disruption to wing loading, tape was used to attach GPS trackers (Falk & Møller, 1995; Nicholls et al., 2002; Phillips, Xavier, & Croxall, 2003; Warham, 1990). GPS trackers were attached to a small group of feathers between the shoulders (2017) or to the three middle tail feathers (2018) using TESA tape (Guilford et al., 2008). Four thin strips of TESA tape were placed under four small sections of feathers (7 × 1 cm tape length), and the GPS tracker was aligned with the antenna directed down the spine. For tail‐fitted devices, three strips of tape were used to attach the GPS units to the tail feathers and the antenna was aligned parallel to the tail feathers. The end of the tape was then folded over the tracker and secured. After securing, any twisted or trapped feathers were repositioned. The combined weight of the GPS attachment with tape (Uria100, 10 g, 2.86%; PinPoint120 standard, 6.1 g, 1.86%) was within 3% of a bird's body weight (~350 g) (Cuthbert, 2001; Warham, 1977).

The Uria100 units were initially set to collect data at 5‐min intervals, which then increased to 15 min. The PinPoint50 GPS trackers used in 2017 were set at 10‐min, 30‐min and 60‐min intervals, whereas the PinPoint120 standard trackers were set at 60‐min intervals. The progression in collection time assisted in maximizing recordings of return foraging trips as battery failure was found to be a problem with these devices. The PinPoint120 Swift Fix units used in 2018 were at 15‐min intervals. To test whether the differences in timing of GPS readings affected the results (e.g., readings every 5 or 15 min vs. every hour), we reanalyzed the tracks of birds using only fixes once per hour. Tracks calculated from hourly fixes underestimated total distance flown by an average of 148.2 km (*SD* = 64.6). As the difference averaged only 12.0% (*SD* = 5.2%) and did not affect the direction of tracks or location of foraging areas, we did not correct for differences in fix rate in subsequent analyses.

TDR loggers were secured to a plastic leg band on the left tarsometatarsus, with the pressure sensor facing toward the foot to limit potential effects of acceleration (Elliott et al., 2008). The TDR loggers recorded pressure (resolution 0.05%), internal device temperature (resolution > 0.05°C), and wet/dry state at 5‐s intervals. TDR loggers were deployed in combination with the PinPoint50 and PinPoint120 Swift Fix GPS units. The combined weight and attachment (PinPoint50, 7.7 g, 2.2%; PinPoint120 Swift Fix, 11.9 g, 3.4%) of each logger combination was within ~3% of a bird's body weight (Cuthbert, 2001; Warham, 1977). Adults were equipped with tracking devices only if their chick weighed over 175 g (to avoid potentially disrupting feeding of hatchlings). Logger deployments were completed between 22:00 and 04:30 hr.

After fitting the TDRs and GPSs, birds were recaptured to recover, download, and redeploy each logger. All recaptures and retrievals of loggers were carried out from 23:30 to 04:15 hr, and each attachment or retrieval took less than ten minutes. In the late evening, each nest entrance was marked with vertical knockdown bamboo pegs (~30 cm), which when displaced indicated the arrival or departure of a bird. Each nest entrance was checked for displaced pegs every 10–20 min. Nests were monitored nightly from approximately 22:00 to 05:00 hr, unless prevented by severe weather. When birds arrived back at their nest, time was allowed for adults to provision chicks, to prevent food loss due to human disturbance. Recaptured birds were examined for signs of damage caused by the TDR units; none was recorded. The TESA tape was peeled off the GPS tracker and removed from the feathers. Few feathers were lost, and no damage or tape residue was detected on the remaining feathers. The TDR logger zip‐tie was cut with scissors, the leg was checked, and no skin abrasion was observed. All birds were released back into their burrows, and the entrance covered for a few minutes to allow the bird to resettle with its chick. Two birds fitted with devices failed to return (one individual in each year). Both were deployed with devices late in the breeding season. The 2017 adult returned early in the 2018 breeding season, and the 2018 bird returned in 2019. The TDR units were recovered from both individuals, but the GPS units had been lost during their molt at sea.

All GPS files were downloaded using preparatory software. We used a remote radio link data transmission system to download the Uria100 data. The base station was installed in the colony and automatically acquired the GPS data each time a bird came within range of the base station (up to 500 m). PinPoint50 and PinPoint120 data were transferred directly to the computer after retrieval from the bird by cable connection.

### 2.2 Data analysis

We tracked a total of 26 foraging trips using GPS and 10 individuals during these deployments were also fitted with TDRs (Table 1). A foraging trip was defined as beginning with the departure of a bird from the colony and ended at its first return to the nest. We visually examined all tracks and defined a return journey as that point at which the bird initiated a track that aligned to the north and thus back to the colony. However, complete tracks were obtained on only 12 trips due to battery depletion and device failure. Data from partial trips were only used to map the outward‐bound flight direction. Maximum distance traveled was calculated from all individuals with complete return journeys. One TDR unit failed to record data due to a computer deployment error while two PinPoint50 GPS trackers returned waterlogged, and seven GPS units were lost at sea. Two birds were not recaptured on initial return and subsequently completed a second foraging trip before the unit was retrieved; only the first GPS track has been used for analysis to avoid pseudoreplication.

All TDR data files were downloaded (Lotek, Tag Talk, Canada) and processed through the program MultiTrace‐Dive (Jensen Software Systems, Germany; version 2014.5.0.0). Dive depth analysis was set to “when wet and ≥1.5 m” to remove TDR manufacturing error (1% error over 100 m = 1.0 m) and barometric pressure influence on the top 0.5 m of water. TDR dive depth and duration data recorded within 15 min of a GPS location (PinPoint50, data collection rate 10–60 min; PinPoint120 Swift Fix and standard, data collection rate 15 and 60 min, respectively) were used to indicate foraging locations. GPS dive durations (preset range 10–60 s) recorded within 15 min of a GPS fix and flight speeds (speed ≤ 10 km/h) recorded by the Uria100 GPS units were also used to indicate potential foraging sites. GPS speeds ≤ 10 km/hr were classed as either foraging, birds taking off, or landing, or resting on the water surface (Kotzerka, Garthe, & Hatch, 2010; Paiva et al., 2010; Weimerskirch, Corre, Ropert‐Coudert, Kato, & Marsac, 2006). The associated Uria100 GPS coordinates and applied equipment limits and the TDR/GPS location data were used to generate kernel density maps (probability level of 95%), allowing us to identify the 50% and 95% core areas used during foraging trips (day 05:00–22:00 hr). Kernel density plots were analyzed by PAST3.10 (Hammer, Harper, & Ryan, 2001).

To determine when birds were inactive for long periods while on foraging trips (i.e., rafting) and the locations of these sites, we used the EMbC package in R to run a Gaussian Mixture Model maximum likelihood estimation algorithm (2017, *n* = 1 individual; 2018, *n* = 5 individuals, 2018; Figure 9 [Garriga, Palmer, Oltra, & Bartumeus, 2016]). The algorithm classifies speed and turning angle of the trajectory to classify behavior. These identified rafting areas were then compared to birds with a GPS fix rate set at 15 min and with flight speeds ≤10 km/hr between the hours of 22:01 and 04:59. Movements below 10 km/hr are likely the result of birds drifting on oceanic currents or blown by winds while sitting on the water surface. An examination of rafting birds fitting this criteria confirmed on average movements of only 1.3 ± 0.1 km/hr (*n* = 378; range 0–8.8 km/hr), supporting the assumption they were largely stationary and not foraging. Birds equipped with TDR devices confirmed there was no nighttime diving activity.

As an initial examination of whether the foraging locations identified by the GPS trackers were related to potential variation in food availability, we used ANOVA to compare differences in chlorophyll *a* concentration and bathymetry between sites with either (a) no dives recorded, (b) one bird diving, or (c) two or more birds diving. Sites were categorized at this coarse level to avoid the pseudoreplication that would result if each dive location were treated separately, since each individual made numerous dives. A random number generator was used to identify 100 sites (at 0.1 min increments) to estimate chlorophyll *a* and bathymetry in areas where no birds were present, and these were then compared to sites where birds were observed foraging. We did not have direct measures of prey abundance across the range of these sites and instead used chlorophyll *a* and bathymetry as surrogates for productivity (i.e., higher regions of chlorophyll *a* and shallower waters would likely contain more prey). The chlorophyll *a* map, in mg/m^3^ concentration (approximately 4 × 4 km; 0.04° spatial resolution), was downloaded as a GeoTIFF raster from Aqua MODIS covering a period of one month (1 January–1 February 2017 and 2018; https://neo.sci.gsfc.nasa.gov), and the New Zealand Bathymetric map was downloaded from ArcMap 10.4 (World Oceans Base Map). Chlorophyll *a* and bathymetric data (NOAA GEBCO; 1‐min bin spaces) were downloaded from NEO NASA Earth Observation as CSV files (https://neo.sci.gsfc.nasa.gov) for analysis.

Pre‐ and postdeployment bird weights, maximum distance from colony, and the total length of foraging trips were tested for normality (Shapiro–Wilk test) and homoscedasticity (Levene's test). Changes in body mass and the interaction between body weight, sexes, and year were analyzed by analysis of variance (ANOVA). Differences in maximum distance from colony and total length of foraging trips and between sex or between years were examined by ANOVA. Subsequent post hoc Tukey's tests for honest significant differences (HSD) were used for multiple comparisons. The significance of the relationship between time and latitude and longitude for the departure and return tracks was assessed using Spearman's rank correlation tests.

Unless otherwise stated, all values are presented as means and ±*SD*. Graphs were produced by Grapher12 (12.5.811).

### 2.3 Ethical statement

This study was performed with permission of the New Zealand Department of Conservation (WAA‐38708‐FAU and WAA‐63957‐RES) and the University of Canterbury Animal Ethics Committee (2014/20R Amendment 2 and 2018/01R).

## 3 RESULTS

### 3.1 Effect of devices on parental condition

Birds weighed on average 350.9 ± 12.7 g (*SD* = 32.3 g, *n* = 25, range 315–435 g) before equipment deployment and 336.2 ± 9.1 g (*SD* = 23.3 g, range 300–400 g) at retrieval. This difference was nonsignificant (*F*_3,23_ = 0.39, *p* = 0.54). The change in weight between deployment and return ranged from +50 to −105 g, but there was no difference between sexes (*F*_3,21_ = 0.02, *p* = 0.90), years (*F*_3,21_ = 0.02, *p* = 0.89), or interaction between sex and year (*F*_3,21_ = 0.41, *p* = 0.53). All chicks successfully fledged from nests in which at least one adult was fitted with a device. Thus, there was no evidence the devices negatively affected the condition of the birds.

### 3.2 Distribution and direction of foraging trips

**Figure 2:**
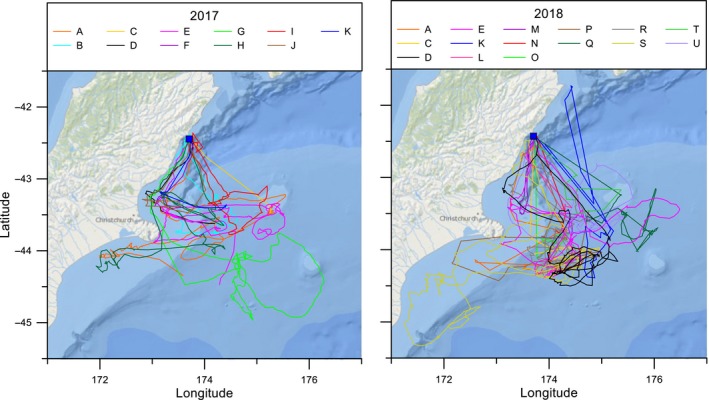
Plot of all fixes for each GPS tracked Hutton's shearwaters during two chick‐rearing periods (11–27 January 2017 and 21 January to 2 February 2018). Different colors used to indicate each bird. Complete return tracks for birds in 2017 (B, H, I, and J) and 2018 (B, D, E, K, L, M, R, S, and U). Partial foraging trips recorded for birds in 2017 (A, C, D, E, F, G, and K) and 2018 (A, C, J, N, O, P, Q, and T). Kaikōura Peninsula colony location is indicated by a solid blue square

**Figure 3:**
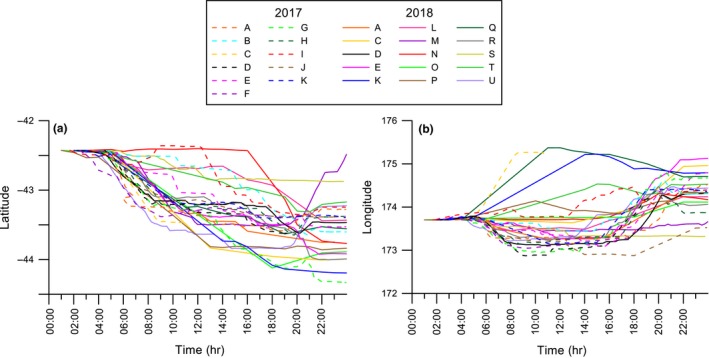
Plot of all outward‐bound tracks recorded over the first 24 hr, independent of date. (a) Latitude by time and (b) longitude by time for 26 Hutton's shearwaters departing the colony and heading south. Individual colors used to indicate each bird during the 2017 and 2018 breeding season

GPS tracks were plotted to show the movements of 26 adults during the chick‐rearing period (Figure 2). Outward‐bound flight paths for 19 individuals were toward the southwest and tracked the coastline, but two birds from this group changed direction and headed southeast after approximately 40 km. Five birds left the colony and flew southeast over oceanic waters. This general pattern of flying south from the colony was evident by plotting the latitude and longitude of each bird during the first 24‐hr period of flight against time (Figure 3). Birds consistently moved to higher latitudes than the colony (Spearman's *r*_s_ = −0.72, *n* = 1760, *p* < 0.001; Figure 3a). During the same timeframe, movement in longitude was variable (Spearman's *r*_s_ = 0.41, *n* = 1,760, *p* < 0.001; Figure 3b) but most birds were observed initially flying west (i.e., tracking the east coast of the South Island), and then veering east.

### 3.3 Near‐shore movements

**Figure 4:**
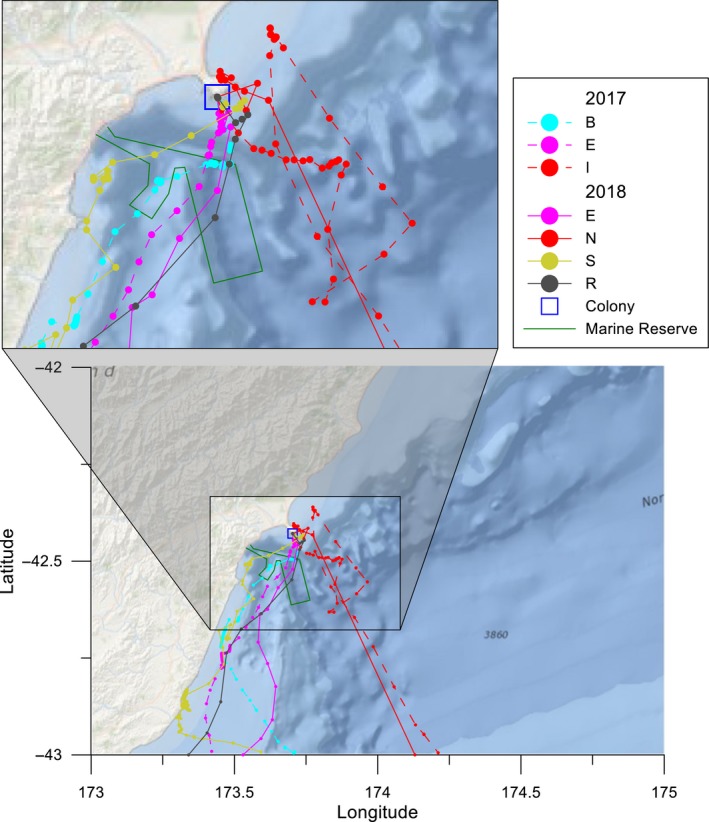
Near‐shore movements of Hutton's shearwaters in the Kaikōura area. Plot of fix locations (circles) and flight tracks for seven outbound birds that spent time within 30 km (zoomed box) of the Kaikōura Peninsula colony during the chick‐rearing period (11–27 January 2017 and 21–25 January 2018). Individual colors used to indicate each bird. The Kaikōura Peninsula colony (hollow blue square) and the Hikurangi Marine Reserve (green line) locations are indicated within the zoomed area

Although the GPS tracks indicate that birds ultimately flew to destinations away from the breeding colony, seven birds spent several hours at the beginning of their trip within the coastal Kaikōura waters before later traveling further away (bird E recorded twice, Figure 4). The time spent by these birds in the Kaikōura area ranged from 1 to 15.3 hr (average 6.4 hr) at distances of 4 to 25 km (average 10.9 km) from the coast. All near‐sea movements began early in the morning (6/7 birds began trips between approximately 04:15 and 05:30 with a single bird beginning at 02:11). Although birds B, E, N, and S spent the most time in proximity to Kaikōura (Figure 4), either no dives were detected (bird E and R 2018) or the birds were not carrying TDRs (birds B, E, I, N, and S).

### 3.4 Trip duration and distance from colony

**Figure 5:**
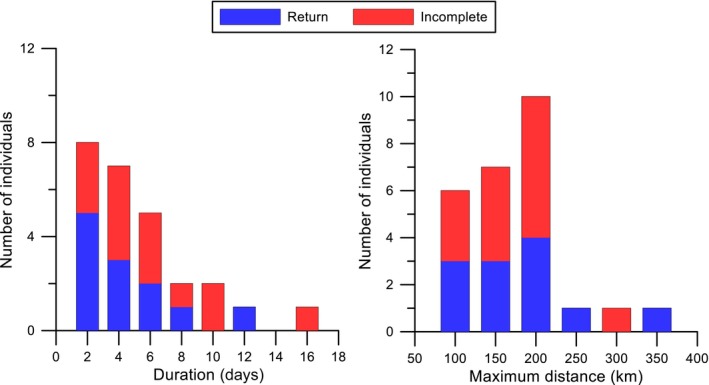
Frequency of trip duration and maximum distance from breeding colony during each trip for Hutton's shearwaters foraging at sea (11–27 January 2017 and 21–25 January 2018). Both completed return trips and incomplete trips are included

The average distance from the colony to the furthest point traveled during a completed foraging trip during the two years of the study was 173 km (2017: range 125–247 km, *SD* = 52.2 km) and 219 km (2018: range 123–365, *SD* = 78.8 km), respectively, and the average total track length was 800.2 km (2017: range 472.3–1,357.6 km, *SD* = 385.9 km) and 1,239.3 km (2018: range 264.7–2,157.2 km, *SD* = 685.8 km). For all birds returning with GPS trackers, the at‐sea foraging trip ranged between 2–15 days (2017: 6 ± 2.5 days; *SD* = 4.2 days) and 1–11 days (2018: 4 ± 1.3 days, *SD* = 2.5 days), respectively. We found no clear evidence of a bimodal foraging pattern (Figure 5).

**Figure 6:**
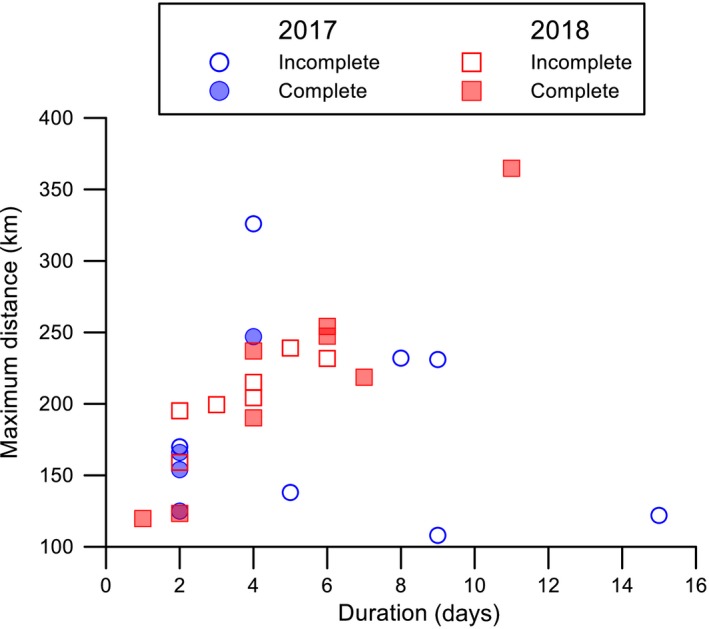
Maximum foraging distances (km) for all recorded foraging trips from the Kaikōura Peninsula in 2017 and 2018, in relation to duration (d) of foraging trip at sea. Symbols indicate incomplete and complete GPS fixes for the return of foraging trips

**Figure 7:**
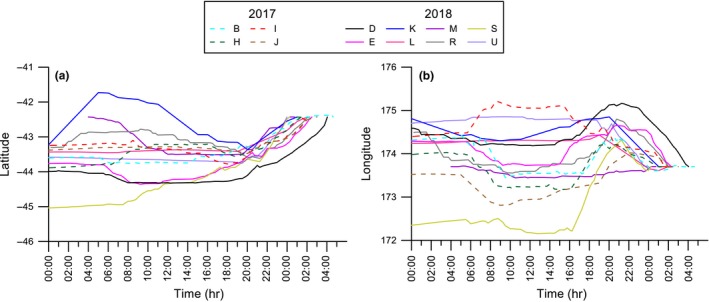
Plot of all inward‐bound tracks recorded over a 32‐hr period for individual birds at sea that completed a foraging trip, independent of date. (a) Latitude by time and (b) longitude by time for Hutton's shearwater adults foraging at a distance from the colony and the return tracks for 12 individuals. Individual colors used to indicate each bird

It might be expected that birds away from the colony for the longest period also flew the furthest (Figure 6). There was a significant positive correlation between duration of the foraging trip and both the maximum distance traveled (*r*_10_ = 0.93, *n* = 12, *p* < 0.001) and the total distance traveled (*r*_10_ = 0.94, *n* = 12, *p* < 0.001). We also tested the relationship between the maximum distance and the total distance traveled from the colony in each year but neither was significant, possibly due to the small sample sizes within a year (2017: *r*_10_ = 0.32, *n* = 12, *p* = 0.32; 2018: *r*_10_ = 0.35, *n* = 12, *p* = 0.27). We also compared the maximum distance from the colony and the total distance traveled by sex, but no significant differences were detected in both cases (*F*_1,10_ = 0.15, *p* = 0.71; *F*_1,10_ = 0.11, *p* = 0.75; respectively). Of the 26 tracked birds, 12 individuals (2017: three females and one male, 2018: three females and five males) completed a return journey over a period of one to eleven days (Figure 7). Individuals started their return trip toward the colony by heading north around 20:00–21:00 hr but due to variation between birds, there was no overall significant change in either latitude (Spearman's *r*_s_ = 0.01, *n* = 1,073 *p* = 0.79; Figure 7a) or longitude during the return journey (Spearman's *r*_s_ = 0.41, *n* = 1,073, *p* = 0.63; Figure 7b). In all cases, return journeys occurred within the last 32 hr of a foraging trip.

### 3.5 Foraging locations

**Figure 8:**
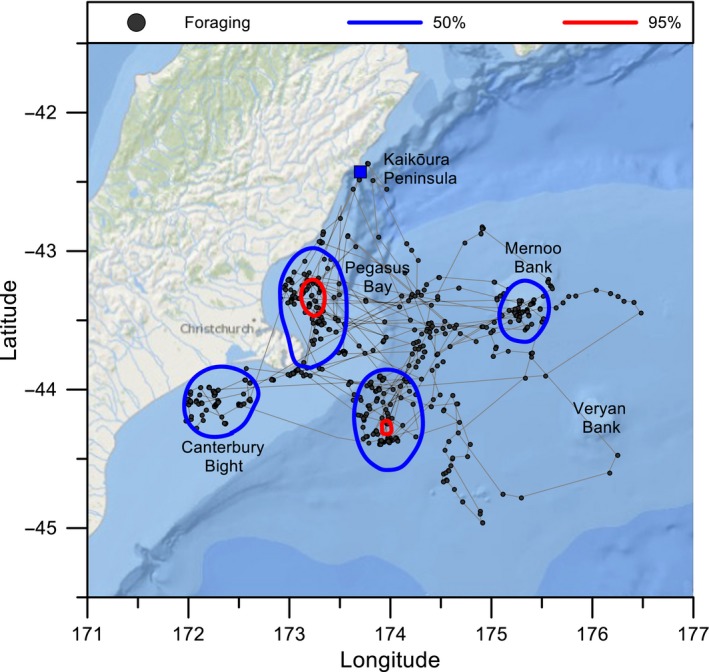
Kernel density plot of diving locations by Hutton's shearwaters. Blue and red ellipses indicate the 50% and 95% kernel utilization distribution of the bird, respectively (grid 4 km resolution). Black dots indicate locations overlaid where birds recorded diving. Kaikōura Peninsula colony location indicated by blue square. Diving identified by TDRs or was defined by birds that were recorded as traveling at speeds <10 km/hr (Uria100 GPS)

Although we cannot rule out that some birds may dive in near‐shore waters, foraging locations were concentrated to the south and southeast of the colony (Figure 8). Four main clusters were identified, two coastal (Pegasus Bay and Canterbury Bight) and two over oceanic banks (Mernoo Bank and Urry Bank). A total of 4,143 dive events were recorded from nine TDR and six URIA100 GPS equipped birds; one of these birds was tracked twice with the two different equipment styles. The maximum depth reached was 30.0 m, and an average maximum depth was 15 ± 2.2 m (*SD* = 5.5 s, *n* = 9 individuals). The average diving depth was 7.1 ± 0.2 m (*SD* = 4.7 s, *n* = 1,454 dives). The maximum and average maximum dive durations recorded were 60.0 s and 46.8 ± 3.9 s (*SD* = 13.4 s, *n* = 14 individuals), respectively. The average diving duration was 23.2 ± 0.3 s (*SD* = 11.4 s, *n* = 4,143 dives; range = 8.1–71.3 s). All dive events occurred between 05:00 and 22:00 hr.

### 3.6 Flight speed

An average flight speed of 10.8 ± 0.4 km/hr was recorded for tracked Hutton's shearwaters (*SD* = 11.8 km/hr, *n* = 3,775; range = 0.1–52 km/hr) when all times were analyzed (i.e., including foraging, rafting, and commuting between locations). During the periods when the bird was defined as either foraging or rafting (i.e., flight speed set at ≤10 km/hr), the average speed of a bird was 1.5 ± 0.1 km/hr (*SD* = 1.7 km/hr, *n* = 2,153). In contrast, the average flight speed when birds were commuting to and from the colony averaged 23.1 ± 0.4 km/hr (*SD* = 7.2 km/hr, *n* = 1,631).

### 3.7 Nocturnal rafting

**Figure 9:**
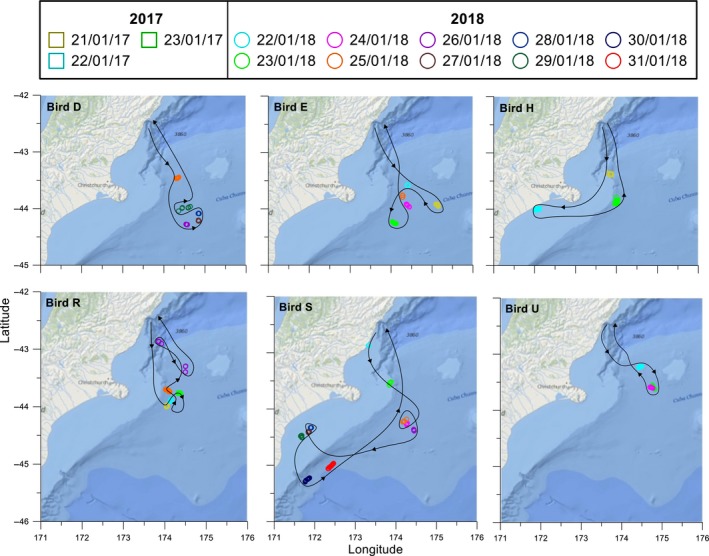
Plot of fix locations of night rafting for five Hutton's shearwater individuals during the 2018 and one during the 2017 chick‐rearing periods. Tightly grouped circles indicate rafting sites. Fix locations recorded between 22:01 and 04:49 hr Flight path indicates the general movement between each night fix site but does not represent the bird's movement during the hours 05:00–22:00

Rafting locations of Hutton's shearwaters while at sea were recorded between 22:01–04:59 hr and occurred over the period of darkness. Little movement was observed except that probably due to drifting on oceanic currents or the influence of the wind. Nighttime rafts were located in a variety of locations, including near the Mernoo and Urry Bank area, within the Canterbury Bight, toward Banks Peninsula and with one individual off the coast of Oamaru (Bird S) (Figure 9). Water depth at rafting sites ranged from 1 m for coastal waters to 1511.8 m deep oceanic waters (average 473.3 ± 65.3 m, *SD* = 299.6, *n* = 81). This was marginally deeper than the sites where one or more birds were observed diving (361.5 ± 105.7 m, *SD* = 400.0, *n* = 55, *F*_1,134_ = 3.47, *p* = 0.06).

### 3.8 Foraging locations in relation to chlorophyll *a* and bathymetry

An ANOVA detected a nonsignificant difference in the chlorophyll *a* concentration between areas in which diving was not observed, or where one or two or more birds dived during the 2017 chick‐rearing season (*F*_2,97_ = 1.43, *p* = 0.24; Table 2). In contrast, a significant difference in chlorophyll *a* values among the 3 categories was detected during 2018 (*F*_2,97_ = 3.97, *p* = 0.02). A significant difference was detected between nondiving areas and where a single bird foraged (Tukey HSD, *p* = 0.02), but a nonsignificant difference was detected in the areas where two or more birds dived and the nondiving areas (Tukey HSD, *p* = 0.15) or between the single bird locations and areas in which two or more birds dived (Tukey HSD, *p* = 0.15). Similarly, a significant difference in bathymetry values was detected for the combined years (*F*_2,97_ = 3.83, *p* = 0.03). A significant difference was detected between the nondiving and areas in which two or more birds dived (Tukey HSD, *p* = 0.02) but a nonsignificant difference was detected between the nondiving and areas where a single bird foraged (Tukey HSD, *p* = 0.91) and between single bird locations and areas in which two or more birds dived (Tukey HSD, *p* = 0.06).

**Table 2:** Summary of randomized plots (*n* = 100) containing chlorophyll *a* and bathymetry values and the presence or absence of diving locations for each tracked bird (mean ± CI; *SD*) during 2017 and 2018 chick‐rearing seasons

Sample	Year	Birds foraging (*n*)	Mean ± CI (*SD*)	Range
Chlorophyll *a*	2017	Absent (63)	0.97 ± 0.12 mg/m^3^ (0.48)	0.44 to 2.65 mg/m^3^
		Single (26)	0.99 ± 0.20 mg/m^3^ (0.51)	0.49 to 2.74 mg/m^3^
		Multiple (11)	1.24 ± 0.28 mg/m^3^ (0.47)	0.65 to 2.07 mg/m^3^
	2018	Absent (77)	0.62 ± 0.05 mg/m^3^ (0.21)	0.30 to 1.41 mg/m^3^
		Single (16)	0.52 ± 0.07 mg/m^3^ (0.14)	0.33 to 0.75 mg/m^3^
		Multiple (7)	0.77 ± 0.05 mg/m^3^ (0.07)	0.65 to 0.83 mg/m^3^
Bathymetry	Combined	Absent (45)	494.3 ± 136.5 m (467.2)	1 to 2,015.8 m
		Single (36)	456.0 ± 142.3 m (435.7)	1 to 1,669.3 m
		Multiple (19)	182.5 ± 109.4 m (243.3)	1 to 724.4 m

Note

Absent, no detected dives; Single, one bird detected in area; Multiple, two to four birds recorded. Chlorophyll *a* concentration and bathymetric depth recorded at a 4 km resolution.

## 4 DISCUSSION

Our results provide some of the first detailed information on the movements and foraging behavior of the New Zealand endemic Hutton's shearwater during the chick‐rearing period. These results demonstrate that Hutton's shearwaters mostly leave the Kaikōura Peninsula colony to forage, often at distances of up to 365 km and remain at sea for up to 15 days before returning to the colony. Foraging areas were concentrated in several regions of high productivity and shallow water. All diving occurred during daylight hours, and birds typically rafted at night in deeper water while on multiday foraging trips. Few birds spent time within the coastal Kaikōura region, showing little evidence of foraging in the area close to the colony. This result was surprising as it had been assumed that most Hutton's shearwaters foraged in the Kaikōura area, or at least much closer to their breeding colonies than we observed in this study (Harrow, 1976; Tarburton, 1981; West & Imber, 1985).

On outbound flights, most Hutton's shearwaters were found to fly south‐southeast from the Kaikōura Peninsula colony. The main direction of travel was along the Kaikōura and Canterbury coastlines toward Banks Peninsula or out over the deep oceanic water toward the Mernoo and Urry Bank areas. Cory's shearwater (*Calonectris diomedea*) use tail and crosswinds to aid in soaring and avoid headwinds (Paiva et al., 2010), but we did not have any data available to test whether variation in the flight paths taken by Hutton's shearwaters was related to wind direction or wind speed. Over the two years of our study, Hutton's shearwaters flew similar flight paths independent of departure date, and the return flight paths of all individuals were reasonably direct, suggesting that any effects of variation in wind direction and speed may have little impact, although this needs to be investigated further.

We found no evidence of a bimodal foraging pattern within the Hutton's shearwater. Some shearwater species (e.g., short‐tailed, sooty, little and Cory's shearwater *Calonectris diomedea*) have been classified as bimodal with foraging trips ranging between short and long durations (1–3 and 5–17 days) (Baduini & Hyrenbach, 2003; Booth, Minot, Fordham, & Imber, 2000; Granadeiro, Nunes, Silva, & Furness, 1998; Shaffer et al., 2009; Weimerskirch & Cherel, 1998). Instead, most trips in Hutton's shearwaters were of short duration (2–4 days), with a gradual decrease in frequency of ever longer duration trips (Figure 5). Long foraging trips may be used by adults to maintain body mass (Baduini & Hyrenbach, 2003), and it has been suggested that a bird's body condition determines the type of trip undertaken (Weimerskirch & Cherel, 1998). The period of the nesting cycle may also affect trip duration as adults may rely on body reserves while foraging closer to the colony in order to provision their chick at more frequent intervals (Cleeland, Lea, & Hindell, 2014; Weimerskirch & Cherel, 1998). As we only tracked Hutton's shearwaters during the nestling period, it is possible that longer duration trips (and thus a bimodal pattern) may be more common at other times of the year or other stages of the nesting cycle.

Our results showed that Hutton's shearwater concentrated foraging in four regions south of the breeding colony, in areas characterized by high chlorophyll *a* and shallower water than nonforaging areas. New Zealand's continental shelf is generally narrow and boarded by extensive submarine plateaus in the northwest and southeast (Leathwick et al., 2006). The Subtropical Front flows around the south of the South Island before heading north along the east coast and out to the Chatham Rise. This area is associated with mixing of subtropical and sub‐Antarctic waters, and high primary productivity (Leathwick et al., 2006). The richest areas of surface water are located over depths of 800–1,000 m, and over the extensive canyon system off the Kaikōura Peninsula. The variable bathymetry in this region is expected to create an area of high productivity, and concentration of prey for foraging seabirds (Bradford, 1972; Mills et al., 2008). It was expected that Hutton's shearwaters would be feeding in areas of high productivity, and therefore, the canyon would be an obvious location. However, this was not observed when we considered chlorophyll *a* concentration levels and bathymetry as indicators for foraging locations. Instead, Hutton's shearwaters foraged further south, though these areas were also characterized by increased concentrations of chlorophyll *a*. Baduini and Hyrenbach (2003) similarly found seabird species with a bimodal pattern of foraging durations were more likely to feed in areas with higher chlorophyll *a* concentration levels within long foraging trips. In contrast, no relationship was detected between primary productivity and foraging location in the black petrel (*Procellaria parkinsoni*) (Freeman et al., 2010). If chlorophyll *a* alone were a proxy for identifying foraging locations in Hutton's shearwater, we would expect more time would have been spent feeding within close proximity to the Kaikōura Peninsula. Instead, we conclude that primary productivity is likely only one factor determining the selection of foraging areas.

In addition to chlorophyll *a* concentrations, we also found that bathymetry may influence foraging behavior as diving locations were concentrated in shallower water than nonforaging areas (Table 2). The shallower waters around coastal areas and over the Mernoo and Urry Banks are associated with eddies and wind‐induced up‐wellings due to the mixing of currents and the variation in bathymetry (Reynolds‐Fleming & Fleming, 2005; Shaw & Vennell, 2000; Vincent, Howard‐Williams, Tildesley, & Butler, 1991). These environmental conditions may provide a more predictable food resource (Phillips, Lewis, González‐Solís, & Daunt, 2017). For example, the black petrel forages in close proximity to the shelf‐breaks along the continental shelf off the North Island (Freeman et al., 2010). Alternatively, sooty shearwater and short‐tailed shearwater prefer colder, deeper, more productive waters which are driven more by oceanic processes (Cleeland et al., 2014; Shaffer et al., 2009). Although further studies of Hutton's shearwater are needed at other times of the breeding cycle, it appears that the underlying bathymetry may play a role in the selection of foraging locations.

We found several rafting locations where birds remained overnight while on multiday foraging trips at sea. Most of these locations were over deep water except for two individuals within the Canterbury Bight and one bird near Oamaru. We recorded twelve return tracks but detected only one individual rafting in the evening near the Kaikōura Peninsula. Rafting behavior in Hutton's shearwater was previously reported in the areas just offshore (~1 km) of Kaikōura, especially during the late evening prior to the birds return to their breeding colonies (Harrow, 1976). As evening approaches, individuals formed large rafts until night‐fall whereby they flew inland *en masse* (Harrow, 1976). These observations probably correspond to the rafting behavior we observed in one individual close to the colony after returning from a more distant foraging trip, although our results also indicate that most Hutton's shearwaters raft at greater distances from the colony than previously suspected.

Hutton's shearwaters are visual predators and were not observed to dive at night (Shoji et al., 2016) and so may need to raft until the morning when they are on multiday foraging trips. However, it is not clear why most individuals appear to raft in slightly deeper waters at night than those locations observed when diving during the day. It has been suggested that Manx shearwaters shift to shallower water at nighttime (Guilford et al., 2008), whereas we found the opposite pattern in Hutton's shearwaters although the pattern was marginally nonsignificant and we observed a high degree of variation in water depths at rafting sites. Breeding Manx shearwaters have also been found to raft at a greater distance from shore than the nonbreeding individuals (Guilford et al., 2008). Perhaps differences in the risk of predation at night or sea surface temperatures may explain this pattern, but there is presently no information to examine either of these possibilities.

Flocks of Hutton's shearwater are regularly seen offshore from Kaikōura, and the belief has been that the birds forage locally within the coastal region (Harrow, 1976; Marchant & Higgins, 1990; Taylor, 2000). Indeed, the recent creation of a marine protected area (MPA) south of Kaikoura was seen as a means of conserving part of the feeding range of this species. In contrast, only seven birds that we tracked remained within close proximity to the colony in our study, and only one bird stopped briefly within the MPA for a short period of time. Similar results were observed for the Scopoli's shearwater (*Calonectris diomedea*), whose distribution at sea also showed little overlap with a previously designated conservation area in Tunisia. In this instance, recommendations were made to extend the marine conservation boundaries (Grémillet et al., 2014). In black‐legged kittiwakes (*Rissa tridactyla*) and Balearic shearwater (*Puffinus mauretanicus*), existing MPAs were found to encompass 50% of the areas around the colonies used for resting and foraging activities, suggesting it is possible to better position protected areas with detailed information of at‐sea behavior (Meier et al., 2015; Ponchon et al., 2017). By identifying areas which are regularly frequented by the Hutton's shearwater, consideration can be made to extend the pre‐existing MPA or to recommend for the establishment of a new MPA area (Taylor, 2000). The current placement of the Kaikōura MPA does not appear to provide sufficient protection for this particular species during the breeding season.

Providing protected areas at sea can be difficult due to the wide range of areas that a species may utilize, and this can be complicated when different areas may be more critical for different age groups (immature vs. breeding birds), breeding stages (prelaying exodus, incubation, chick‐rearing), or foraging trip type (short or long), or when the selection of foraging locations is affected by irregular climatic events (e.g., El Niño and La Nina). As the marine environment is subject to increasing pressure from fisheries, tourism, and deep‐sea oil exploration, it may become impossible to safeguard such wide‐ranging species as Hutton's shearwaters across large areas of their range. Instead, ensuring that human use of the marine environment throughout the range of the Hutton's shearwater is done in such a way that minimizes by‐catch and does not deplete prey populations may be a better strategy for their conservation than relying solely on the creation of protected areas.

## CONFLICT OF INTEREST

None declared.

## AUTHORS' CONTRIBUTIONS

The study was designed jointly by all authors. DGB and LR undertook the field‐work and collection of data. DGB carried out the data analysis and writing. TWH, SJG, and JVB supervised the study and contributed to the writing.

## Data Availability

Data supporting this study are available from the Dryad Digital Repository (https://doi.org/10.5061/dryad.r492c47).
